# Exclusion of actin microfilaments from the cell-cell contact zone in HTLV-1 infected T-lymphocyte during the establishment of a functional virological synapse

**DOI:** 10.1186/1742-4690-8-S1-A199

**Published:** 2011-06-06

**Authors:** Mohamed Nejmeddine, Isabelle Clerc, Graham P Taylor, Charles R M Bangham

**Affiliations:** 1 Department of Immunology, Wright-Fleming institute, Norfolk Place, Imperial College London, London, W2 1PG, UK; 2 Department of Medicine, infectious diseases group, Norfolk place, Imperial College London, London,W2 1PG, UK

## 

Human T-lymphotropic virus type-1 (HTLV-1) spreads efficiently between T-cells via a tight and highly organized cell-cell contact known as the virological synapse (VS), by analogy to the immunological synapse. It is now clear that other retroviruses, including the human immunodeficiency virus type-1 (HIV-1) murine leukemia virus (MLV), also spread efficiently by cell-to-cell contact via a virological synapse.

The polarization of the microtubule-organizing center (MTOC), in the infected cell toward the target cell, is an important feature of the establishment of a functional HTLV-1-VS. This polarization is induced by a synergistic combination of signals from HTLV-1 Tax protein within the cell and from cross-linking of adhesion molecules at the cell surface. However, the mechanism by which the virions are delivered to the VS and the role of other component of the cytoskeleton are still not completely understood.

We have investigated the chronology of events following the polarization of HTLV-1 proteins (Gag, Env and Tax) in the donor cell toward the target T-cell, and the concomitant changes in the actin microfilaments. We show that the presence of Gag and Env at the VS is accompanied by exclusion of the actin microfilaments at the zone of the cell-to-cell contact, whereas Tax protein did not exclude actin. These results suggest that during the establishment of a functional VS, rearrangement of the actin microfilaments in the infected cell facilitate the transfer of HTLV-1 virions between cells.

